# Did flowering plants exist in the Jurassic period?

**DOI:** 10.7554/eLife.43421

**Published:** 2018-12-18

**Authors:** David Winship Taylor, Hongqi Li

**Affiliations:** 1 Department of BiologyIndiana University SoutheastNew AlbanyUnited States; 2 Department of BiologyFrostburg State UniversityFrostburgUnited States

**Keywords:** *Nanjinganthus*, fossil, Jurassic, paleobotany, angiosperms, flower, Other

## Abstract

The discovery of a fossil that might be the oldest flowering plant will continue the debate on the origin and structure of ancestral flowering plants.

**Related research article** Fu Q, Diez JB, Pole M, García Ávila M, Liu Z-J, Chu H, Hou Y, Yin P, Zhang G-Q, Du K, Wang X. 2018. An unexpected noncarpellate epigynous flower from the Jurassic of China. *eLife* **7**:e38827. doi: 10.7554/eLife.38827

"This shouldn't be here," said Ellie, the paleobotanist in *Jurassic Park*, as she stared at a leaf. "This species of veriforman has been extinct since... the Cretaceous period." Ellie might have been equally impressed and surprised if she had stumbled across a fossil recently discovered in China that appears to be of a flowering plant that dates to the Jurassic period.

One of the characteristics of flowering plants is that they produce seeds within an ovary or carpel, which is why they are also called angiosperms (*angio*-, container; *sperms*, seeds). They became widespread during the Cretaceous period, and now come in about 300,000 different species and dominate most landscapes. Models based on comparisons of plant DNA changes over time (Magallón et al., 2015) and a study of geochemical biomarkers by the present authors and others (Taylor et al., 2006) suggest that angiosperms originated before the Cretaceous period (which started 145 million years ago), and perhaps even before the Jurassic period (which started 201 million years ago). However, unequivocal fossil evidence of angiosperms only dates back to 135 million years ago, well after the end of the Jurassic period. Moreover, recent reports of Jurassic flowers by Xin Wang of the Nanjing Institute of Geology and Palaeontology and co-workers (which are summarized in Wang, 2017a; Wang, 2017b) and pollen have not been widely accepted (see Herendeen et al., 2017 for a review).

Now, in eLife, Xin Wang, Zhong-Jian Liu of the Orchid Conservation and Resesarch Center of Shenzhen and international co-workers – including Qiang Fu as first author and co-workers in Spain, Australia and other institutes in China – report evidence for an angiosperm from the Early Jurassic (Fu et al., 2018). They base this claim on the fact that this new species, which they have called *Nanjinganthus*, possesses the characteristics of the earliest angiosperms as published by Bateman, Hilton and Rudall in 2006 (Bateman et al., 2006). Here we discuss whether or not *Nanjinganthus* fulfills our criteria to be considered a Jurassic angiosperm, including whether it possesses the structural features that we would expect to find in an ancestral angiosperm.

First, the age and dating of the fossiliferous sediment must be reliable, and the fossils should be collected in situ by the researchers to ensure dependable placement and stratigraphy. Fu et al. collected numerous specimens of *Nanjinganthus* from localities with strong biostratigraphic dating to the Early Jurassic, so the finding passes this test. Second, the fossil species must have at least one agreed-upon defining characteristic (such as an ovary); moreover, any additional characteristics must not be a defining characteristic for any other group of living or fossil non-flowering seed plants (which are collectively known as gymnosperms). *Nanjinganthus* does exhibit strong evidence that the seeds are within an ovary, which falls within a rather narrow definition of an angiosperm (Wang, 2017a; Wang, 2017b), and Fu et al. conclude that the other characteristics of their specimens do not define any gymnosperm. Third, the fossil should have multiple characteristics of an angiosperm, and many of these should be consistent with the ancestral characteristics put forward by other researchers, based on studies of well-preserved fossils and modern plants. Given that Fu et al. discuss *Nanjinganthus* with respect to only a limited number of these characteristics, here we explore this criterion in more detail.

First, we examined the ancestral characteristics predicted by Peter Endress and James Doyle in 2009, based on a phylogenetic analysis of basal living angiosperms (Endress and Doyle, 2009). Based on our interpretation of the fossil, we found that 23 of 29 floral characteristics (79%) preserved in *Nanjinganthus* matched the predictions (Figure 1).

**Figure 1.**
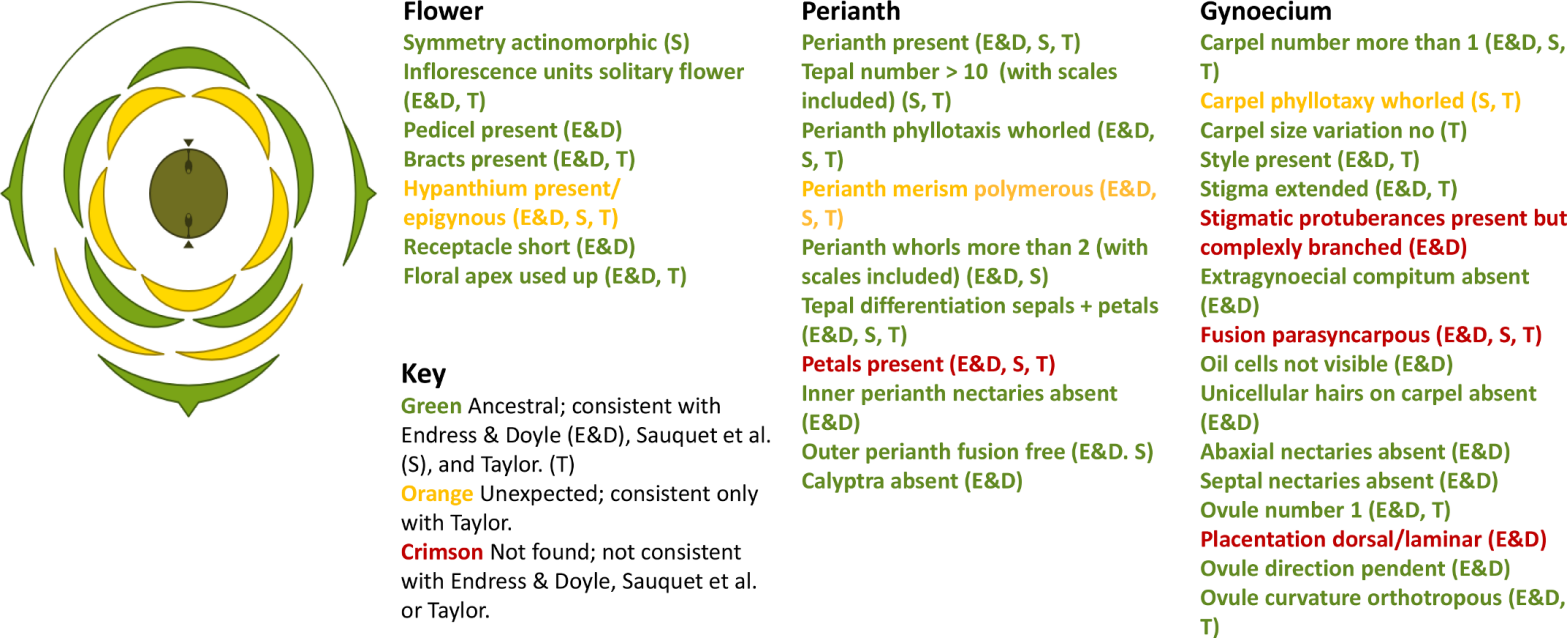
Characteristics of an ancestral flowering plant. Floral diagram of the Jurassic fossil *Nanjinganthus* (left), and the characteristics for the flower, perianth and gynoecium of the ancestral flowering plant as predicted by Endress and Doyle (E&D), Sauquet et al. (S), and Taylor (T). The floral diagram is our interpretation of the fossil and it shows (starting at the outside): one bract and two fused bracteole (green); two scales (orange); a whorl of five sepals, which could be four (green); a second whorl of five petals, which again could be four (orange); five fused carpels (which could be four) forming an inferior ovary/epigynous flower (olive green and black) that contains two ovules/seeds (black triangles) that are attached to the outer walls of the ovary; it is also possible for there to be one or three ovules/seeds. The characteristics of *Nanjinganthus* shown in green in the three lists are consistent with Endress and Doyle, Sauquet et al., and Taylor; the characteristics shown in orange are only consistent with Taylor, and those shown in crimson are not consistent with any of these three sources. Floral diagram created with the Floral Diagram Generator at http://kvetnidiagram.8u.cz/odiagramech_en.php

Second, of the 13 characteristics predicted by the evolutionary-developmental model of Hervé Sauquet and co-workers (Sauquet et al., 2017), we found six (46%) in *Nanjinganthus* (Figure 1). However, the fossil has seven characteristics that were not predicted by either model. For example, the fossil has a complexly branched style/stigma attached on top of the ovary, surrounded four or five petals, and the seeds are attached on the middle of the carpel walls (Figure 1). Finally, we examined a suite of better-preserved, Early Cretaceous fossil species previously summarized by one of us (Taylor, 2010) and we found 15 of the 18 characteristics (83%) in *Nanjinganthus* (Figure 1). The only characteristics not found in these fossils were the presence of petals, fused ovary, and seed attached on the middle of the carpel wall (presence of branched style/stigma was not reported).

From this analysis, we infer that *Nanjinganthus* shows substantial similarity to predicted models of ancestral characters and Early Cretaceous angiosperms, so the evidence suggests that it is a Jurassic flowering plant. *Nanjinganthus* is clearly an important fossil, but additional characteristics need to be documented, the similarities to angiosperms need more careful justification, and its relationships to other species should be analyzed phylogenetically. Finally, the Jurassic angiosperms previously reported by Wang and co-workers could be reevaluated with our criteria to assess if they are missing angiosperms. New fossils and additional analyses will finally confirm the presence of angiosperms in the Jurassic period and strengthen our understanding of the ancestral angiosperm.
